# Controlling Cytomegalovirus: Helping the Immune System Take the Lead

**DOI:** 10.3390/v6062242

**Published:** 2014-05-27

**Authors:** Patrick J. Hanley, Catherine M. Bollard

**Affiliations:** Program for Cell Enhancement and Technologies for Immunotherapy, Sheikh Zayed Institute for Pediatric Surgical Innovation, and Center for Cancer and Immunology Research, Children’s National Health System, Washington, DC 20010, USA; E-Mail: CBollard@childrensnational.org

**Keywords:** Cytomegalovirus (CMV), Adoptive immunotherapy, T cell, immunotherapy, cellular therapy, transplant

## Abstract

Cytomegalovirus, of the Herpesviridae family, has evolved alongside humans for thousands of years with an intricate balance of latency, immune evasion, and transmission. While upwards of 70% of humans have evidence of CMV infection, the majority of healthy people show little to no clinical symptoms of primary infection and CMV disease is rarely observed during persistent infection in immunocompetent hosts. Despite the fact that the majority of infected individuals are asymptomatic, immunologically, CMV hijacks the immune system by infecting and remaining latent in antigen-presenting cells that occasionally reactivate subclinically and present antigen to T cells, eventually causing the inflation of CMV-specific T cells until they can compromise up to 10% of the entire T cell repertoire. Because of this impact on the immune system, as well as its importance in fields such as stem cell and organ transplant, the relationship between CMV and the immune response has been studied in depth. Here we provide a review of many of these studies and insights into how CMV-specific T cells are currently being used therapeutically.

## 1. Biology of Cytomegalovirus

The virus family *Herpesviridae* consists of three subfamilies of viruses, alpha, beta, and gamma. Betaherpesviruses contain the four major components of this family: the core, the capsid, the tegument, and the envelope and establish latency in cells of the myeloid lineage and CD34+ cells [1,2,3]. The tegument contains the majority of virion-associated proteins [4,5]. Tegument proteins have two reported functions, though the proteins that facilitate them are not mutually exclusive [6]. The first function is the disassembly of the virion during entry and assembly of the virion during egress [7]. The second function is inhibiting the host immune response to infection, though as discussed later, they may also promote the response as well [6]. Many of these proteins are associated with immune evasion, and are therefore packaged within the virion and delivered to the host soon after uncoating of the virus. The most abundant tegument protein is the lower matrix phosphoprotein of 65 kDa (pp65), or Unique Long (UL)83 [5]. One important function of pp65 is immune evasion. Other tegument proteins devoted to immune evasion include the upper matrix protein pp71, UL36, UL38, and IRS1/TRS1 [8,9].

Immediate early (IE) proteins are translated within 2 h of infection and do not require the de novo synthesis of viral proteins for their translation [10]. These IE proteins then control subsequently gene expression and virus replication. As such, suppression of IE proteins is thought to contribute to CMV latency whereas the expression of IE genes is associated with reactivation [11]. The laboratories of both Hahn and Fietze have shown that proinflammatory cytokines such as GM-CSF and TNF-α can induce the differentiation of monocytes into macrophages or dendritic cells, which is thought to activate the IE1 promoter and stimulate reactivation [12,13,14]. However, how cytokines cause HCMV reactivation is still mostly unknown [12,14]. Because IE proteins are expressed first after reactivation, T cells targeting these proteins are of paramount importance, as highlighted in the field of transplantation where T cells IE proteins are important for protection after solid organ transplant [15].

## 2. CMV Infection in the Immune Compromised Host

CMV has long been one of the most problematic pathogens after stem cell transplantation (SCT) and organ transplant [16,17,18,19]. While effective antiviral drugs, viral monitoring, and donor/recipient matching have lowered the likelihood of disease after SCT, the mortality rate in patients who develop CMV-associated pneumonia remains strikingly high (around 80%–90%) [17,18]. Additionally, the recipient’s CMV-seropositivity remains an independent risk factor for morbidity and mortality after SCT. In the case of SCT, the highest risk of CMV reactivation is when the recipient is seropositive and the stem cell donor is seronegative [20,21]. This is because the recipient has latent (or active) CMV that can no longer be controlled by the recipient’s immune system after it is depleted with conditioning regimens and the stem cell donor graft does not contain protective CMV-specific memory T cells. In contrast, the risk of CMV-related complications, including death, after solid organ transplant (SOT) is greatest when the organ donor is CMV-seropositive and the recipient is CMV-seronegative, though the severity tends to vary based on the organ being transplanted [22,23,24,25]. With the advent of CMV prophylaxis, an unexpected complication has emerged with an increased incidence of late-onset CMV disease; after day 100, late CMV disease may be as high as 17% in CMV-seropositive recipients undergoing SCT [26].

## 3. Innate Immunity to CMV

An in-depth review of the immune response to CMV can be found here [9]. Some of the best evidence for the role of the innate immune system in mice is in experiments using beige mice that have known defects in Natural Killer (NK) cell-mediated cytotoxicity and are highly susceptible to murine Cytomegalovirus (MCMV). However, protection against MCMV can be restored by transferring NK cells from normal mice [27]. Despite the elegant studies suggesting the importance of NK cells in controlling MCMV, similar studies in humans are lacking for HCMV [28]. However, Biron *et al*. have reported in the New England Journal of Medicine NK cell-deficient individuals who are susceptible to herpes virus infections, including HCMV. In vitro experiments using IL-2 activated human NK cells have also demonstrated that NK cells can inhibit CMV replication in CMV-infected fibroblasts by inducing IFN-beta release from infected fibroblasts [29]. Activated NK cells also released IFN-gamma which can impede viral replication [9]. Boehme *et al*. have shown that HCMV glycoproteins B and H also activate Toll Like Receptor (TLR) 2 on fibroblast, resulting in NFκB activation and subsequent inflammatory cytokine secretion, suggesting that NK cells are not the only innate cell responsible for protection from CMV [30]. Indeed, monocytes, macrophages and dendritic cells are cells permissible for viral reactivation and once infected release inflammatory cytokines, in addition to presenting antigen to T cells [31].

## 4. Humoral Immunity to CMV

The importance of an antibody response to CMV is demonstrated in guinea pig models where antibodies protect the animals from reaching a lethal infective dose, but do not prevent infection, suggesting a role for the humoral immune response in limiting the severity of the disease by controlling CMV viral load [32]. After a primary infection in humans, antibodies against a number of proteins from HCMV are detectable in the serum. These antibodies recognize an array of proteins from different parts of the virus, including pp65 and pp150 from the tegument, the glycoproteins gB and gH from the envelope, as well as proteins involved in transcription such as IE-1 [33]. Most CMV-seropositive humans have antibodies directed against gB, with over 50% of all neutralizing antibodies recognizing an epitope of gB [33,34]. The importance of humoral immunity is also demonstrated in congenital CMV infection where pregnant women who develop primary CMV infection carry a 40% chance of HCMV transmission to the fetus [35,36]. In cases where the mother is able to provide transplacental IgG antibodies, the severity is less [37]. Moreover, identifying a successful vaccine that elicits functional neutralizing antibodies to CMV and can prevent congenital CMV has become a priority [40]. Relevant articles discussing the role of the humoral immune response can be found here [39,40,41].

## 5. Cellular Immunity to CMV

**Figure 1:**
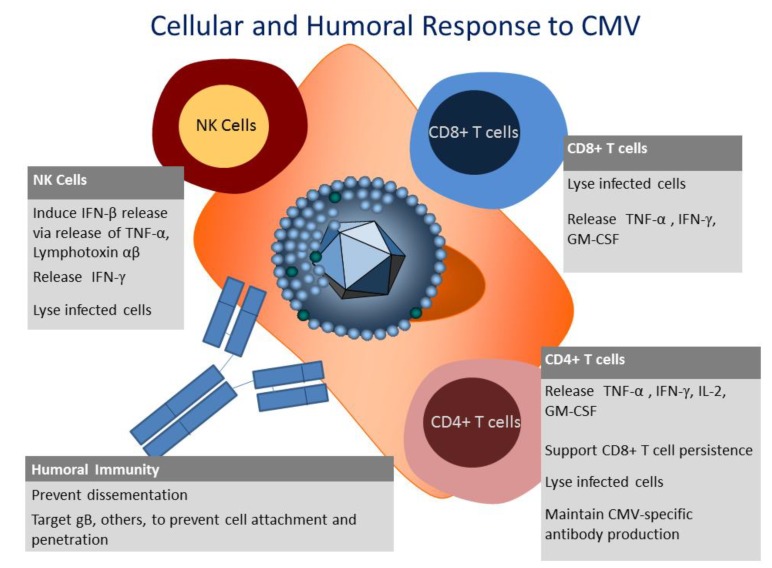
Cellular and humoral immunity to Cytomegalovirus.

CMV infection commands an overwhelming response from all facets of the immune system. As discussed above, the humoral response and innate response to CMV are indeed significant and contribute to controlling the infection, but the cellular immune response is necessary to control latency and impede viral replication in latently infected individuals (Figure 1) [42]. The most compelling evidence for the immunogenicity of CMV involves the cellular arm of the immune system where up to 10% of all circulating CD8+ T cells can be directed towards CMV—a staggering number given the plethora of pathogens we encounter in our lifetime [43]. With the extraordinary percentage of T cells targeting CMV, it has been postulated that over time, immune surveillance is less effective in CMV+ individuals and, although controversial, CMV-seronegative individuals have been reported to live longer than their CMV-seropositive counterparts [44]. Recent reports on the “aging” of the immune response to CMV have begun to shed light on the mechanism behind this observation [45,46].

## 6. CD8+ T Cell Response to CMV

The most studied, understood, and manipulated facet of the immune response to CMV is the cellular response, in particular the protective role of CD8+ T cells. The presence of CMV-specific T cells was first reported in a SCT study noting that the majority of patients without CMV-specific T cells had overwhelming CMV disease [47]. This led to studies evaluating the *ex vivo* expansion of CMV specific T cells from CMV-seropositive individuals [48] and the extensive evaluation of the CMV-specific T cell response in healthy individuals *versus* immune compromised populations. The importance of CD8+ T cells has been reported in solid organ transplant as well [49]. In renal transplant recipients, the presence of CD8+ T cells coincides with protection from CMV [50,51,52] and in both heart and lung transplant recipients, the presence of IE-1-specific T cells correlated with protection from CMV disease [15].

The protectiveness of CD8+ T cells was first shown in a study by Riddell *et al*. where T cells were expanded *ex vivo* using CMV-infected fibroblasts [48]. Expanded T cells were then infused into patients after SCT. Riddell *et al*. reported that prior to the infusion of the T cells, the CMV-specific immunity was undetectable in these patients. However, as early as 48 h after infusion there was a detectable CMV-specific response and after three weeks the CMV-specific T cells response was as great or greater than CMV-specific immune responses detected in immunocompetent donors [48,53].

The cell mediated immune response targets proteins in all three of these stages of the viral replication cycle: immediate early, early, and late. By targeting immediate-early and early genes, T cells can protect against reactivation from latent viruses [54]. Although targeting late proteins would typically delay the cellular response to CMV since these proteins are not expressed until 24-h after infection, many of the late proteins are structural proteins such as the tegument protein pp65 which is already present within the virion and can be immediately presented to T cells by infected APCs.

In spite of the plethora of CMV antigens targeted by the CD8+ T cell response, a hierarchy of antigenic frequency exists, though it varies on the method of detection. Collectively, pp65 and the immediate-early 1 (IE-1) are two of the most targeted antigens of CMV by CD8+ T cells [55,56].

## 7. CD4+ T Cell Response to CMV

As is the case with CD8+ T cells, approximately 9% of all circulating CD4+ T cells recognize CMV, an astounding number given the immune repertoire devoted to protection from one pathogen [56]. These T cells recognize at least 125 open reading frames of CMV or 59% of all open reading frames. Antigen recognition by CD4+ T cells is similar to CD8+ T cells in that T cells recognizing pp65 are some of the most abundant but T cells recognizing other antigens such as gB, UL86, pp28, IE-2, UL36, UL48, pp10, UL113, and IRS-1 are also highly abundant [56].

The importance of CD4+ T cells is apparent in a model of CD4-T cell-depleted mice where mice infected with MCMV had an increased incidence of MCMV reactivation [57]. It has also been reported that CD4+ T cells contribute to controlling MCMV in mice depleted of CD8+ T cells, but viral clearance is significantly delayed in most tissues and is never cleared from the salivary glands where a persistent infection develops [58].

The significance of CD4+ T cells is better understood and supported in HCMV. Lung transplant recipients with low frequencies of CMV-specific CD4+ T cells have difficulty clearing CMV [49]. In a parallel to the finding in mice, children with CMV who have few CD4+ T cells have prolonged shedding of CMV in the salivary glands, and dysfunctional CD4+ T cells have been reported during primary infection [59,60]. Studies from SCT recipients suggest that CD4+ T cells are linked with protection from disease and are necessary for the recovery of donor-derived CD8+ T cells [61]. What is perhaps more interesting is that the persistence of adoptively transferred T cells has been reported to depend on the presence of CD4+ T helper cells [53].

In the first study of adoptively transferred CMV-specific T cells (predominantly CD8+), no patients developed CMV viremia but only patients who had detectable CD4+ T cell responses showed persistence of the transferred CD8+ T cells [53]. In other words, the highly successful trials mentioned above that utilized CD8+ T cells specific for various antigens of CMV required not only the transferred CD8+ T cells, but endogenous CD4+ cells as well.

A compelling argument for the role of CD4+ T cells in immunity to HCMV derives from a study conducted by Einsele *et al*. in which CD4+ T cells (without CD8+ cells) were transferred to antiviral-resistant SCT recipients with CD4+ T cell deficiencies. Remarkably, all patients exhibited rapid antiviral activity and the cells then persisted at levels similar to immunocompetent healthy donors. Einsele *et al*. observed that the presence of CD4+ T cells allowed endogenous CD8+ CMV-specific T cells to expand, in contrast to transferring CD8+ T cells that eventually decline without CD4+ T cell help [61].

## 8. CMV Evasion from the Immune System

Through thousands of years of evolution, CMV and humans have reached a balance whereby CMV is able to transmit virus from host-to-host yet not cause significant pathology to immunocompetent individuals. One way CMV is able to persist is by employing numerous immune evasion genes that are expressed in both the unique short (US) and unique long (UL) region of the genome (Table 1) [62]. As with all herpes viruses, CMV interferes with MHC class I presentation to CD8+ T cells in a number of ways. Interestingly, CMV also appears to stealthily modulate the immune response to itself by using decoys and choosing which epitopes and antigens it allows the immune system to target. More specifically, phosphorylation of IE-1 by pp65 blocks the processing of IE-1 in the proteosome [63]. This evasiveness is likely a reason why IE-1 was not identified earlier as an important immunogen of CMV.

**Table 1:** CMV genes involved in immune evasion.

Mechanism of evasion	CMV Gene product	Effect on immune system
MHC Class I down-regulation [64]	US2, US3, US6, US11	Decreased presentation of CMV antigens to CD8+ T cells
CMV-IE-1 sequestration [63]	UL83 (pp65)	T cells cannot target first genes expressed upon reactivation
MHC Class II down-regulation [65,66,67]	IE/E product	Decreased presentation of CMV antigens to CD4+ T cells
MHC Class I homolog [68]	UL18	Inhibition of NK cell lysis
Inhibitory receptors, downregulation of ligands [69,70]	UL40, UL16, UL142	Evasion of NK cells
Chemokine receptor [71]	US28	Immune homing interference
IL-10 homolog [1]	UL111a	Immune suppression
Inhibitors of apoptosis [72,73]	UL36, UL37	Decrease in phagocytosis of infected cells by APCs
Downregulation of MICB expression [74,75]	MicroRNAs (miR-UL112)	Decreased recognition by NK cells and T cells via NKG2D

Another way CMV disrupts antigen presentation is by disrupting the transporter associated with antigen processing (TAP). US6 binds with high affinity to the ER-lumenal side of the transporter, effectively altering the affinity of TAP for ATP. Overall, at least four US genes are involved in down regulation expression of MHC Class I: US2, US3, US6, and US11 [64,76].

Dendritic cells play an important role in the primary immune response to CMV as they orchestrate the priming of naïve T cells in the lymph nodes. Not surprisingly, CMV targets dendritic cells and halts their maturation, forcing them into a state of functional paralysis and preventing them from presenting CMV antigens to T cells [62,77]. What’s more unexpected is that DCs infected with MCMV are not just unresponsive to MCMV, but MCMV-infected DCs do not secrete IL-12 or IL-2 even after treatment with the potent stimulus lipopolysaccharide (LPS) [77]. It has been suggested that CMV not only evades the immune system, but it also suppresses it. Secondary CMV-associated diseases exist and are a result of CMV modulation of the immune response in ways mentioned above. Additional immunomodulatory genes expressed by CMV and their function are listed in Table 1, but for a more comprehensive review of immune evasion by CMV see the reviews cited here [78,79,80].

## 9. Immunotherapy: Adoptive Transfer of CMV-Specific T Cells after Transplant

As discussed above, some of the most insightful data about the role of T cells in protection from CMV came from adoptively transferring T cells to patients who received stem cell transplants. Since the first method of generation CMV-specific T cells that was utilized almost 20 years ago by Riddell *et al*. using CMV-infected fibroblasts to expand T cells, numerous other methods have been developed to offer protection after SCT [81]. The most common and perhaps most simple is pulsing antigen presenting cells—typically dendritic cells—with overlapping peptides spanning the entire pp65 antigen [82]. Although efficacious, this technique requires the lengthy and difficult generation of DCs, a non-trivial amount of donor-derived blood, and requires highly trained technicians and expensive equipment and manufacturing facilities.

In 2006, Leen, Bollard, and Rooney *et al*. generated T cells specific for three viruses (CMV, EBV, and adenovirus) by using a recombinant adenovirus that expressed pp65, hereafter named Ad5f35pp65 [83]. This approach utilized monocytes and EBV transformed LCL transduced with the Ad5f35pp65 vector as APCs, thus obviating the need for high volumes of donor blood and targeted 3 viruses in a single culture. While this strategy was effective *in vivo* and less labor intensive, the expansion of virus-specific T cells is still a lengthy process that takes upwards of one month, not including the months it takes to generate EBV-LCL [83].

To circumvent the need for multiple expansions and a lengthy expansion process, Peggs *et al*. recently described a method where they pulsed leukocytes with overlapping peptides for pp65. Activated T cells are then selected based upon their secretion of IFN-gamma and then frozen for infusion. Including QA/QC testing, total time to infusion was less than two weeks and the entire selection process took <24 h. However, the risk of such a rapid manufacturing strategy is GVHD since alloreactive T cells may be still present in the infused product. Indeed, in the Peggs study, most patients were protected from CMV at a dose of only 1 × 10^4^ CD3+ cells/kg but 8 of 18 patients developed acute GvHD, three of which were grade II or higher. Six patients also experienced chronic GvHD [84].

Memory T cells are critical in non-T cell depleted grafts because the virus-specific memory T cells present in the grafts confer protection against viral infections and reactivation. For this reason, CMV-reactivation is highest when the transplant recipient is CMV-seropositive and the transplant donor is CMV-seronegative, as is often the case in cord blood transplantation and CMV-seronegative donors [85]. Memory T cells are also a valuable resource when expanding virus-specific T cells *ex vivo* from seropositive donors as these T cells can be expanded by simply culturing virus-specific memory T cells with antigen-presenting cells loaded with the antigen of interest. When transferred to transplant recipients after transplant, these cells have been protective against CMV, EBV, and adenovirus without severe adverse events (Table 2) [83,86]. The challenge, however, has been the *ex vivo* generation of antigen-specific T cells from antigen-inexperienced sources of T cells such as cord blood. Instead of expanding the pre-existing memory T cell population, naïve T cells need to be primed *in vitro* to respond to the antigen of interest [19].

**Table 2:** Studies of adoptively transferred CMV-specific T cells.

Group	Method of Expansion/Selection
Riddell, 1992, 1995 [48,53]	Expansion using CMV-infected fibroblasts
Einsele, 2002 [61]	Expansion with CMV lysate
Cobbold, 2005 [87]	Tetramer Selection using magnetic beads
Leen, 2006 [83]	Antigen-presenting cells (Dendritic cells, EBV-LCL) transduced with an adenoviral vector encoding CMVpp65
Micklethwaite, 2008 [86]	Antigen-presenting cells (Dendritic cells) transduced with an adenoviral vector encoding CMVpp65
Peggs, 2011 [84]	Selection of T cells secreting IFN-γ after exposure to CMV antigen
Hanley, 2012 [88]	Antigen-presenting cells (Dendritic cells, EBV-LCL) transduced with an adenoviral vector encoding CMVpp65
Blyth, 2013 [89]	Antigen-presenting cells (Dendritic cells) transduced with an adenoviral vector encoding CMVpp65 or Dendritic cells pulsed with HLA-A02-restricted peptide NLVPMVATV

Because of these challenges, only a few reports document the generation of single antigen-specific T cells from naive donors [90,91,92,93]. In an attempt to target 3 viruses simultaneously, our group reported the ability to generate CMV, EBV, and adenovirus-specific CTL from the 20% fraction of a cord blood unit by using dendritic cells transduced with an Ad5/f35-CMV-pp65 vector as well as the cytokines IL-7, IL-12, and IL-15 [94]. Responding T cells were shown to be derived from the naïve T cell population and responded to typical and atypical, novel CMV-pp65 epitopes. A clinical trial using CB-derived multi-virus specific T cells for the prevention and treatment of viral infection after CBT is open and has started to accrue patients. (Clinical Trial #: NCT01017705) [81,88]. Recently, we and other groups have also reported the ability to generate CMV-specific T cells from CMV-seronegative donors; [95,96] the clinical efficacy of these T cells will be tested in a Phase 1 clinical study (Clinical Trial #: NCT01945814).

Another option for recipients of CB and CMV-seronegative donors is the use of third party, CMV-specific T cells. Thirdy party virus specific T cells have been evaluated clinically in several trials with promising results [97,98,99,100,101]. Leen *et al*. recently published a multi-institutional study of best-matched, MULTI virus-specific T cells and reported that responses with third party CTL were similar to those from donor-derived CTL in their previous studies [83,100]. This study highlights the importance of epitope recognition when selecting the optimal third party T cell lines.

## 10. Summary

The human body has done an exquisite job over thousands of years to prevent or impede the manifestations of CMV and as a result, the majority of the population can live with CMV without knowing they have it. However, when the balance shifts towards CMV reactivation, usually as a result of treatment modalities or in some cases other infections, options are available to treat CMV infection, and the use of immunotherapy is rapidly becoming one of the favored options. The use of third-party, epitope-targeted CMV-specific T cells provides a unique platform similar to other pharmacotherapies in that they are rapidly available, are short lived, are effective, and are not associated with significant toxicities. However, for chronically suppressed patients, such as those undergoing SOT, the use of autologous CMV-specific T cells might be the ideal solution as long-term protection is necessary. Overall, improvements to T cell manufacturing technologies will provide a new and widely used treatment for CMV infection after transplant.
